# Roux-en-Y Gastric Bypass and Heller Myotomy: One-Step Surgical Treatment of Symptomatic Achalasia in a Morbid Obese Patient

**DOI:** 10.1007/s11695-021-05376-z

**Published:** 2021-04-29

**Authors:** Nunzio Velotti, Antonio Vitiello, Giovanna Berardi, Mario Musella

**Affiliations:** Department of Advanced Biomedical Sciences, University of Naples “Federico II”, Via Pansini n.5, 80131 Naples, Italy

**Keywords:** Achalasia, Bariatric surgery, Roux-en-Y gastric bypass, Heller myotomy

## Background

Achalasia is a rare disorder of esophageal motility characterized by the absence of peristalsis and incomplete lower esophageal sphincter (LES) relaxation during swallowing. It usually presents with progressive severe dysphagia and its most effective treatment is surgical disruption of the LES with a Heller myotomy (HM) [1]. Obesity is an independent risk factor for esophageal motility disorders, which may exist in >50% of morbidly obese patients [2]. However, obesity is not generally associated with achalasia and the prevalence of this disease in obese patient population varies from 0.5 to 1% [3]. Bariatric surgery currently represents the best treatment option for morbid obesity and its related diseases and laparoscopic Roux-en-Y gastric bypass (LRYGB) is the second most commonly performed procedure in the world [4]. At same time, HM has demonstrated its superiority over other treatments for achalasia [5]. However, resolution of achalasia symptoms with only laparoscopic HM, expose an obese patient to the risk for further weight gain. Even though the separated management of the two pathologies is well described, current literature lack of consistency about a simultaneous treatment [6, 7].

## Methods

Here we present a rare case of a 46-year-old patient with a Body Mass Index (BMI) of 47 kg/m2 and an intractable achalasia diagnosed by a high-resolution esophageal manometry (HRMi) and x-rays of upper digestive tract showing aperistalsis and dilation of the esophagus with failure of LES relaxation. Patient had already undergone two endoscopic pneumatic dilations over the 3 years prior to presentation with temporary efficacy.

## Results

**Fig. 1:**
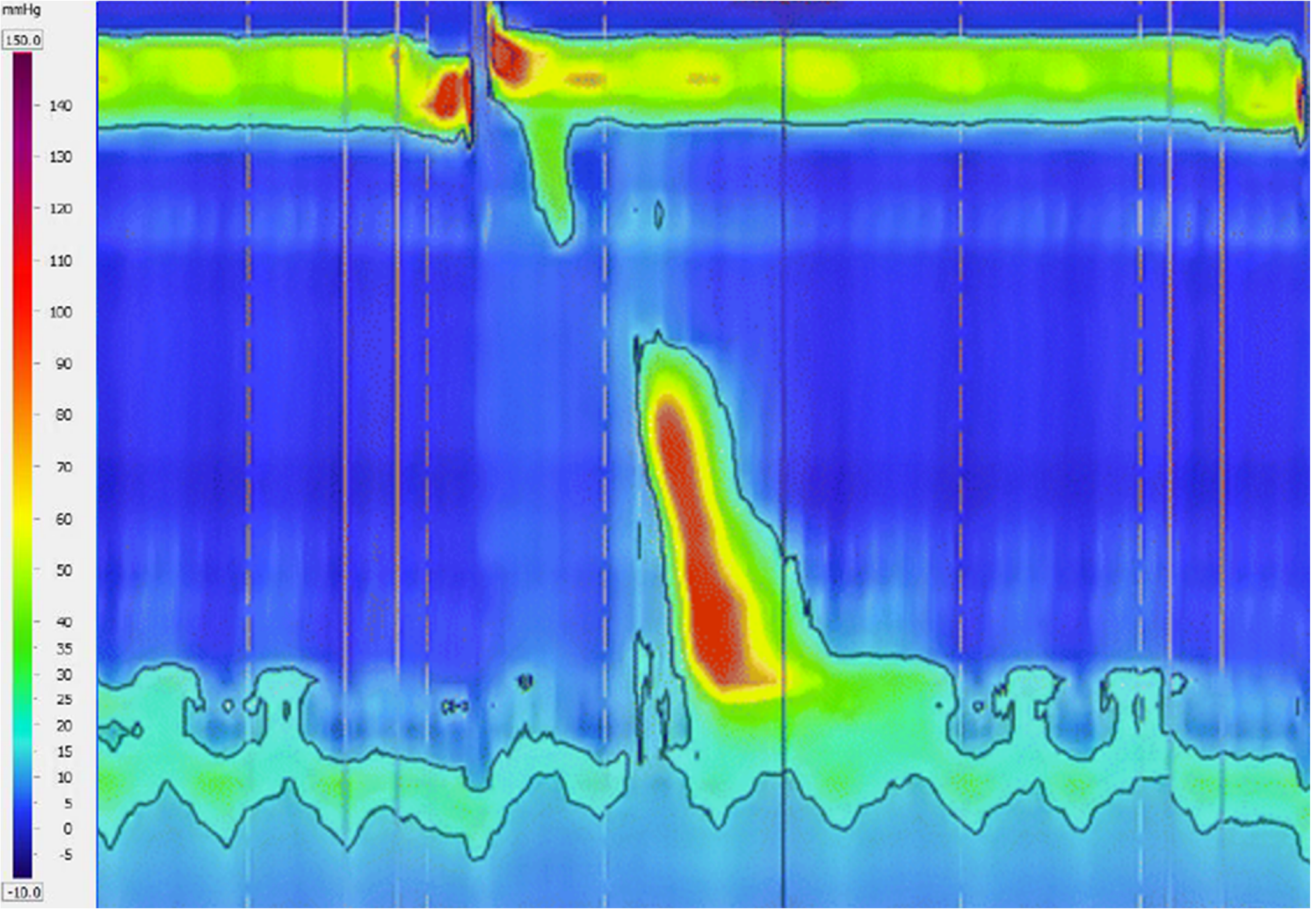
Six months follow-up high-resolution esophageal manometry (HRMi)

He underwent concomitant laparoscopic HM and LRYGB and 6 moths follow-up demonstrated a reduction of BMI to 34,7 kg/m2 and the remission of esophageal symptoms. (Figure 1) The patient was classified with an ASA (American Society of Anesthesiologists) score 3 and did not have any previous surgery. Operative time was of 115 minutes and no intraoperative complications were recorded. On post-operative day (POD) 3 the patients started a liquid diet; on POD 5 he was discharged. No early (<30 days) complication occurred.

## Conclusion

With the increasing spread of obesity worldwide, the combination of these two diseases may became more frequent and our report suggest thatsimultaneous surgical management of achalasia and obesity with a minimally invasive approach (Heller myotomy and RYGBP) is feasible with successful results for both weight loss and esophageal dysmotility.

## Supplementary Information

ESM 1 (MP4 374118 kb)
